# An experimental study of information transparency and social preferences on donation behaviors: the self-signaling model

**DOI:** 10.3389/fpsyg.2023.1258808

**Published:** 2023-11-10

**Authors:** Minnie H. C. She, Alan G. Sanfey

**Affiliations:** ^1^Behavioural Science Institute, Radboud University, Nijmegen, Netherlands; ^2^Donders Institute for Brain, Cognition and Behaviour, Radboud University, Nijmegen, Netherlands

**Keywords:** charity donation, prosocial behavior, dictator game, information seeking, social preference

## Abstract

Certain segments of the population reply on charitable or other non-governmental organizations as their main source of support, with these organizations largely funded by those in society who can afford to give. The present study investigated to what extent information transparency influences donation decisions, and whether specific preferences for charities influences information seeking behavior. We recruited 114 participants via Prolific and employed a binary online Dictator Game to address these two study objectives. The results showed that participants’ actual donation behavior was not influenced by their charity preference or the level of information transparency. However, they were more prone to seek out additional information when deciding about the most preferred category of charity. These results raise important questions as to whether the perceived anonymity of online choices may differ from choices carried out in person.

## 1. Introduction

Are human beings prosocial? To a certain extent the answer to this seems to be “yes,” as research has often shown that people do tend to be concerned about the welfare of others and, when given the opportunity, are willing to sacrifice some of their own gains for the greater good. Yet, it remains somewhat unclear why people would choose to help others (e.g., donating money to charity) given typically finite resources. Are all good deeds backed by inherent kindness, or rather are there other, hidden, agendas behind these seemingly other-regarding actions? This study explores the respective roles of personal preference and situational circumstance on prosocial behavior.

Donation behaviors are often considered as prosocial. However, prosocial behaviors need not to be altruistic (Pfattheicher et al., 2022). Depending on the intention, whether people are aiming to help others to meet their needs or to obtain some form of self-gratifying rewards, their prosocial behaviors can be driven by altruism or egoism (Batson et al., 2011). Considering the diverse motivations of behaviors, in this study, we take a broad definition of prosocial behaviors, focusing on the behavior itself in order to examine how information transparency influences people’s donation decisions, and how preferences for charitable categories drive information seeking.

### 1.1. Dictator game and donation behaviors

In the decision science literature, the Dictator Game (DG; Kahneman et al., 1986) has been frequently adopted to investigate how people weigh personal gain vs. another’s gain under diverse conditions. This social dilemma therefore provides a concrete basis to carefully explore situational circumstances that may give rise to prosocial behaviors, such as donations to charity. A typical DG is comprised of a “Dictator” (i.e., the test participant) who is tasked with making decisions about how to divide an experimentally-provided amount of money (e.g., 10 euros) between themselves and a passive “Recipient.” The recipient has no say in how the money will be distributed; it is solely the decision of the dictator. Interestingly, contrary to the suggestions of most traditional economic models that Dictators will retain all of the money for themselves, the majority of people who play the role of the Dictator are not completely egoistic and selfish. A meta-analysis of 131 papers (616 treatments) showed that, on average, dictators give 28.35% of their endowments to the recipient(s) (Engel, 2011). Moving beyond grand means reported from these treatments, Engel (2011) also reconstructed 20,813 individual observations from 328 treatments that provided complete distribution information. From these reconstructed observations, the majority of the dictators (around 64%) gave at least a portion of their endowments to their recipient(s), with approximately 17% of the dictators giving half of their endowments away and nearly 5.5% of them giving all away.

Economic games, like the Dictator Game, allows control and manipulation of specific factors to study their influence in isolation on behaviors. Its nature as laboratory experiment sometimes raise questions on the external validity of the findings – do people behave similarly in real life as in the laboratory, when stakes are usually higher? In order to address contextual influences in DG, some studies manipulated the source of endowment (Cherry et al., 2002) or the recipient (Eckel and Grossman, 1996), aiming to mimic characteristics of situations where people distribute resources between themselves and others. Indeed, when people were given the option to share money earned in the experiment (vs. money that handed over to them), they are less altruistic (Cherry et al., 2002). This perhaps can explain why average households donated roughly 0.4% of their total household expenditure to charity (McKenzie et al., 2011) as the donations were taken directly from their hard-earned money. Nonetheless, considering that donation behaviors in the lab and in the field were positively correlated (Eckel and Grossman, 1996), this offers some level of confidence over findings derived from experimental setups.

### 1.2. Self-signaling model: who are we as a person?

When we act upon our decisions, we are giving out signals about who we are as a person through our action or inaction (Bodner and Prelec, 2003). The self-signaling model suggests that by reflecting on these signals, we can examine our own self-image for identity discrepancies (Bodner and Prelec, 2003). Bodner and Prelec (2003) divided the self into two subtypes with different, sometimes contrasting, concerns and priorities. First, there is the decision-maker self that focuses on their material well-being and, perhaps less so, the well-being of others (Grossman and van der Weele, 2017). This part of the decision-maker self is modeled as outcome utility, looking at the consequences of our choices and their associated practical values (Bodner and Prelec, 2003). Second, the observer self aims to minimize the discrepancy between the desired self-image and the actual image (Feiler, 2014). Thus, it gathers diagnostic information from our decisions, checks the alignment between the ideal and perceived self, and then adjusts self-perception on aspects of our self-image accordingly. This is modeled as diagnostic utility, helping to achieve alignment. Put simply, the observer self-interprets one’s own actions from an outsider perspective, then evaluates these actions at face value without assessing the underlying considerations.

In everyday life, decision-making involves some competition between these two utilities. For example, where X is a course of action, Y is the outcome, and Z is the desired self-image, the dilemma would look like: “Doing X would lead to outcome Y. However, if I did X, would I think less of myself as being Z?” When the subjective values of diagnostic utility outweigh outcome utility, people will choose the course of action that helps reinforce their perceived self-image as opposed to the action that maximizes benefits. In the context of the Dictator Game, when a person is presented with a fair and an unfair option for distributing a portion of money between themselves (as the decision-maker) and another person (as the passive recipient), if the decision-maker places a high diagnostic utility on perceiving themselves as prosocial (i.e., Z), in spite of the values they placed material gain (i.e., Y) and knowing that the unfair option (i.e., X) maximizes their material gain, it is likely that they will pick the fair option. In other words, with reference to the self-signaling model, it is possible for a person to compromise their material gain in order to maintain their positive self-image as prosocial.

### 1.3. Incomplete information and willful ignorance

When a situation offers sufficient flexibility for an individual to maximize gain while preserving their positive self-image, they may choose differently. This kind of flexibility (i.e., moral wiggle room) can be influenced by numerous factors, and thus situational ambiguity offers easy routes for people to justify or rationalize their otherwise immoral behaviors. Under situational ambiguity, normative expectations on behaviors are loosened when insufficient information is available to discern the right course of action to take. This condition expands the (moral) wiggle room that people are usually afforded.

Whereas some may pursue full information, better informing themselves about what normative expectations to adhere to, others may take advantage of this opportunity to wiggle out of the norms by being willfully ignorant. By this, people can actively shy away from the opportunity to obtain complete information, thus avoiding potential intrapsychic dilemmas between potential actions. An experiment conducted by Dana et al. (2007) tested this by experimentally creating situations where people had the choice to willfully ignore complete information. Dana et al. (2007) introduced a modified version of DG with binary choices, in which one option contained an equal allocation of money (i.e., $5 for dictator, $5 for recipient) and the other contained an unequal, self-benefiting, allocation of money (i.e., $6 for dictator, $1 for recipient). They also added a twist in an incomplete information condition, manipulating information transparency by using a “?” to mask the allocation of money to the recipient instead of showing the exact amount. Dictators could choose to either reveal the exact allocation before they made their decision, or to remain ignorant. The results showed that participants tended to choose the equal option in the complete information condition (74%), where the payoffs for both participants and recipients were transparent. This is consistent with previous findings in the DG, as outlined above. However, this percentage dropped significantly when participants were not initially presented with the exact allocation to recipients (28% chose equality). Despite being economically costless, only 56% of participants chose to reveal the true payoffs.

These results suggested that the generous sharing behaviors observed in the complete information condition, as well as in classic DG experiments, could be a form of socially desirable action to meet others’ and one’s own expectations. Indeed, in the transparent condition, some people found it difficult to convince themselves that choosing the self-benefiting option is “moral” because the direct (negative) consequence to recipients is obvious and clearly linked to their own actions. However, the incomplete information condition provided some “wiggle room” for them to act as if they are ignorant, even though this ignorance was self-imposed, allowing them to interpret the available information in ways that were the most personally beneficial (van Baar et al., 2019).

### 1.4. Social preferences and ingroup favoritism

Whilst some participants were self-serving, others chose to reveal the exact allocation and acted consistently with their prosocial decisions. This could be fueled by social preferences. Depending on who is the recipient, hence the perceived social closeness with the recipients, people could decide to distribute resources between themselves and others differently. Referencing to the social identity literature (e.g., Tajfel, 1978, 1982; Tropp and Wright, 2001; Balliet et al., 2014), people tend to differentiate between “us” and “them.” After self-categorizing into social groups, the categorization would initiate positive thoughts and behaviors associated with the ingroup, in order to differentiate it from outgroups (Tajfel, 1978, 1982). When considering some people, groups, or issues as part of our ingroup identity, we tend to subconsciously feel closer to and are more willing to benefit these groups, even extending positive affection, moral regard, and benevolence toward these groups and individuals (Graham et al., 2017). Thus, when making a decision that influences individuals or groups whom we feel close to or identify with, we could be more prosocial.

### 1.5. About the study

Using the self-signaling model as its core theoretical framework, the present study aims to understand the effect of situational characteristics and personal preference on donation decision. The experiment by Dana et al. (2007) serves as the cornerstone of the current study to explore further: (1) the effect of information transparency on donation behaviors to the most and the least preferred charitable categories; and (2) whether charity preference would influence information seeking behavior, and subsequently the donation decision.

Based on this theoretical background, the first research question is to what extent does information transparency influence donation decisions directed to different charitable categories (RQ1). With reference to the self-signaling model, we hypothesize that participants are less likely to choose the prosocial option in the incomplete information condition as compared to the complete information condition (H1a). Additionally, considering that individuals have personal preferences for different charitable categories, some of these recipients would be situated closer to the self in the participants’ moral circle than others. We hypothesize that participants will choose the prosocial option more for the most preferred charitable category than the least preferred charitable category (H1b).

The second research question is to what extent charity preference influences information seeking behavior and the subsequent donation decision (RQ2). With reference to self-signaling theory, people should sort themselves into different actions based on personal preferences, and therefore we hypothesize that participants are more likely to seek complete information about money allocation for the most preferred charitable category than the least preferred charitable category (H2a). Furthermore, we hypothesize that participants who actively seek complete information are more likely to choose the prosocial option than those who deliberately remain ignorant (H2b).

## 2. Materials and methods

### 2.1. Participants

The data were collected via Prolific^1^. Based on the results from a previous study (Dana et al., 2007), a power analysis was conducted with G*Power 3.1 (Faul et al., 2009), which suggested that 108 participants would be sufficient to detect medium effect (α = 0.05, effect size = 0.27, 80% statistical power). A total of 120 participants were recruited, and completed the experiment. Six participants were removed as they finished the online experiment in an exceptionally quick time (i.e., 6 min or less, approximately half of the mean completion duration). The final sample therefore had 114 participants (Male = 51, Female = 63; *M*_age_ = 34.20, *SD* = 13.91, range = 18–75). A sensitivity analysis was conducted via G*Power (Faul et al., 2009). It indicated that with *p* = 0.05 (two-tailed), and a power of β = 0.80, our final sample can provide sufficient statistical power to detect a minimum effect size of odds ratio = 1.98 (for selfish trials, Model 1, 3, and 4) and odds ratio = 0.50 (for prosocial trials, Model 2 and 5) for the main effects of information transparency and charity preference.

Participants were compensated with £1.25^2^ (approximately equivalent to €1.95) for completing the experiment. Additionally, before starting the experiment, they were told that there was a possibility for them to earn a bonus payment based on both the decisions they made during the experiment as well as luck (i.e., a random event). The maximum possible bonus, depending on condition, ranged from €1.50 to €2.50. The exact amount of the potential bonus was not disclosed to participants. On average, participants took 11.6 min to complete the whole experiment, and earned €0.78 as a bonus.

### 2.2. Experimental design

#### 2.2.1. General procedures

The experiment consisted of three parts^3^ : (1) a questionnaire about personal preferences regarding charitable organizations; (2) a filler task to prevent carryover effects on awareness of one’s charity preference on performance in the subsequent experimental task; and (3) a modified-dictator game to assess social decision-making. The instructions used in the experiment are included in the online Supplementary Appendix.

#### 2.2.2. Questionnaire

The questionnaire was designed to assess the personal importance of different social causes. This was measured by asking participants to rate five different categories of charitable organizations based on how important they felt each category was to them (1 = Not at all important; 7 = Extremely important). The five categories were dog rescue/re-housing, education in disadvantaged communities, environmental protection, a mental health helpline, and support for homeless individuals. Next, participants were instructed to rank the five categories based on personal importance, prioritizing first the charitable category that they considered the most important. This task gave participants the opportunity to evaluate and differentiate their subjective levels of urgency/preference for each charitable cause. In addition, the age and gender of participants were collected as demographic data.

#### 2.2.3. Filler task: remote associates task

In order to lessen participants’ potential self-consciousness about their charitable attitudes and feelings toward charitable organizations, and its impact on their performance in the upcoming donation-related experimental task, eight trials of the Remote Associates Task (RAT; Mednick, 1962) were placed between the questionnaire and the experimental tasks to act as a filler. In a trial of RAT, participants were first presented with three clue words. Then, they were instructed to type a fourth word that connected with the three words. For example, if the three probe words shown on the screen are “white,” “scramble,” and “shell,” a possible answer for the fourth word is “egg.” Each trial had a time limit of 15 s. Responses from this task were not included in subsequent analyses.

#### 2.2.4. Modified dictator game

In the modified dictator game (DG), participants were required to make choices between an equal or an unequal distribution of resources between themselves and a charity (Dana et al., 2007). Participants always played the role of the decision-maker, and completed four blocks of trials, two blocks each for the complete and incomplete information conditions (both are discussed in detail below). Each block consisted of 10 trials.

**FIGURE 1:**
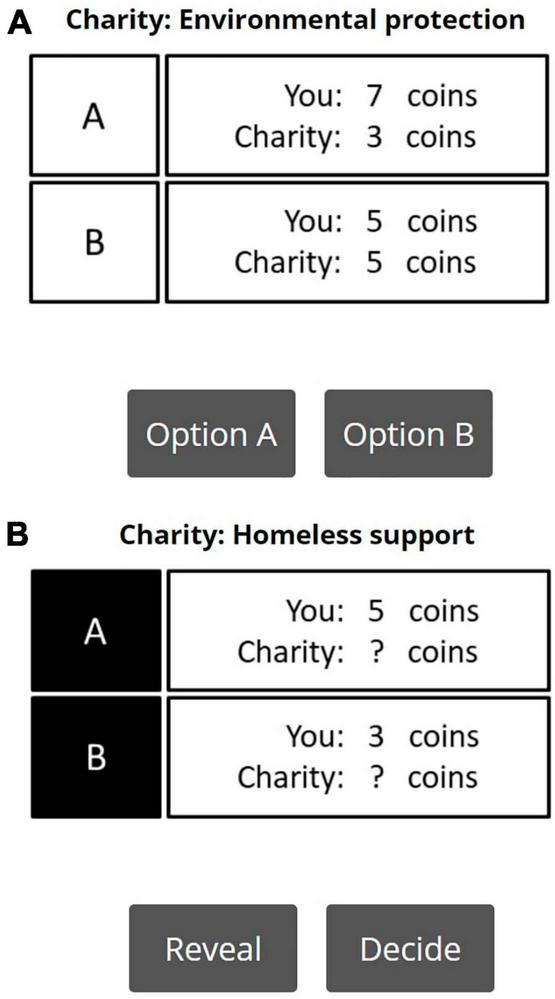
**(A)** An example trial in the complete information condition. Participants are fully informed of the distribution of the resources of the two options. **(B)** An example trial in the incomplete information condition. The distribution of resources of the two options only partially shown initially. Participants can choose to obtain full information by clicking the ‘Reveal’ button. Alternatively, they can make their choice right away, without knowing the distribution by clicking the ‘Decide’ button.

There were two within-subject manipulations used in this modified DG. First, the experiment adapted two conditions from Dana et al. (2007) to manipulate information transparency (i.e., complete vs. incomplete information). This manipulation aims to give the power to participants to choose whether they want to expose themselves to information that potentially leads to a dilemma between material gain and positive self-image. In the complete information condition, participants were presented with two options: one option proposed an equal split of 10 coins (i.e., five coins for the participant and five coins for a charity), while the other option proposed an unequal split. The degree of inequality in the latter option ranged from completely benefiting the charity (i.e., 0 coins for the participant and 10 coins for a charity) to completely benefiting the decision-maker (i.e., 10 coins for the participant and 0 coins for a charity). Table 1 showed the unequal options per trial used in the experiment (while the equal option is constant with a 5 to 5 split). The same 10 decision trials were presented to participants in randomized order. Participants chose between the two options by clicking one of the two buttons (i.e., A or B). An example of a complete information trial is shown in Figure 1A. The presentation of the equal and unequal options was counterbalanced, meaning that the equal option was assigned to Option A half of the time, and to Option B in the other half. Notably, in this condition the information about coin distribution is completely transparent.

**TABLE 1:** Trials on splitting the coins in complete vs. incomplete information condition in the experiment.

	Complete information		Incomplete information	
**Trial^a^**	**Participant**	**Charity**	**Participant**	**Charity**
1	10	0	10	0
2	9	1	9	2
3	8	2	8	2
4	7	3	7	1
5	6	4	6	4
6	4	6	4	8
7	3	7	3	7
8	2	8	2	3
9	1	9	1	1
10	0	10	0	4

^a^The trial number is for descriptive purpose only. In the experiment, trials were presented to participants in randomized order in each condition.

In the incomplete information condition, participants were similarly presented with two options. However, here only partial information about the distribution of coins was provided. For instance, all trials in this condition initially only showed the amount that the decision-maker would receive but left ambiguous the amount that would be sent charity (see Figure 1B). Participants were informed that there was a 50 percent chance that the total number of coins that would be split was 10 (i.e., the same number as the complete information condition), and that there was a 50 percent chance that the total number of coins was randomly determined (i.e., the amount for charity would be independent of the amount that the participant gets).

While the participants were blind to the exact distribution of coins by default, they were given an opportunity to reveal the proposed allocation of coins if they pressed the “Reveal” button on the screen. If a participant pressed the “Reveal” button, the complete information of the distribution of coins for that trial was displayed on screen for them to consult before choosing between the two options. Participants were neither instructed to click on the “Reveal” button, nor penalized for choosing not to reveal the actual coin distribution. It was instead entirely up to the participants if they wanted to make the allocation decision either fully informed or without knowing the distribution of coins (i.e., being willfully ignorant). After reading the instruction, there was a step-by-step walk-through to demonstrate the consequences of selecting either the “Reveal” or “Decide” options (see Supplementary Appendix 1). Again, the assignment of the equal and unequal splits to Option A or Option B was counterbalanced. The order of trials was randomized.

The second within-subject manipulation concerned the personal importance of charitable categories, aiming to direct donation decisions to categories that fell within different moral circles of the participants. Each participant therefore saw both their most preferred and least preferred charitable categories, according to the category rankings filled out by participants in the questionnaire. The complete and incomplete information conditions each contained one block directing decisions to the most preferred charitable category of the participant, and one block to the least preferred charitable category.

Across all 40 trials in both conditions, one trial was randomly selected in order to implement the bonus payment. The experimental coins were multiplied by a factor of 0.15 to convert into Euros. Based on the chosen allocation of coins in the randomly selected trial, the monetary amounts were paid to participants as a bonus payment and donated to the respective charitable category, if applicable.

We disclosed all measures, manipulations, and exclusions in the studies. Full materials, scripts and datasets are available at https://osf.io/73h5q/.

## 3. Results

The data analyses were conducted using R (R Core Team, 2022). For hypotheses that used equal-unequal decisions as the dependent variable, each were fitted with two mixed-effect models – one for trials that contained an equal option and an prosocial option (i.e., allocation to charity > self, hereafter referred to as prosocial trials), and another for trials that contained an equal option and a self-benefiting option (i.e., allocation to self > charity, hereafter referred to as selfish trials). As the prosocial and selfish trials would result in different response patterns, conducting the analyses separately can avoid canceling out any potential effect (Table 2).

**TABLE 2:** Distribution of equal-unequal decisions: information transparency conditions by charity preferences.

	Most preferred charitable category				Least preferred charitable category			
	Selfish trials^+^ (*n* = 570)		Prosocial trials^#^ (*n* = 570)		Selfish trials^+^ (*n* = 570)		Prosocial trials^#^ (*n* = 570)	
	% of choosing equal option	% of choosing selfish option	% of choosing equal option	% of choosing prosocial option	% of choosing equal option	% of choosing selfish option	% of choosing equal option	% of choosing prosocial option
Complete information	283 (49.6%)	287 (50.4%)	280 (49.1%)	290 (50.9%)	319 (56.0%)	251 (44.0%)	267 (46.8%)	303 (53.2%)
Incomplete information	276 (48.4%)	294 (51.6%)	303 (53.2%)	267 (46.8%)	301 (52.8%)	269 (47.2%)	287 (50.4%)	283 (49.6%)

**^+^**trials that contain an equal option (5 for participant; 5 for charity) and a selfish option (e.g., 10 for participants; 0 for charity); **^#^**trials that contain an equal option (5 for participant; 5 for charity) and an prosocial option (e.g., 0 for participants; 10 for charity).

Five binominal mixed-effect models were run using the function *glmer* from the lme4 package (Bates and Maechler, 2015). Given that each participant gave multiple responses in the experiment, to account for this non-independence, participants were included as a grouping variable in the models. The random structure consisted of a random intercept for participant and random slopes were specified based on each model (Table 3). Random covariance was removed for all, except Model 5, to resolve singularity warnings when fitting. Additionally, in order to support convergence, the number of coins allocated to participants and charity were standardized, charity preferences were centered, and sum-to-zero contrasts were used for the factor of reveal-not reveal decision (omitted group: not reveal). All models were run with *bobyqa* as the optimizer, except for Model 3 that used *Nelder_Mead* as the optimizer due to singularity warning. *P-*values were determined using the function *mixed* from the package afex (Singmann et al., 2019; version 0.23.0), used type 3 tests and the method of Likelihood Ratio Tests. Confidence intervals were obtained using the built-in function *confint* in R, and the method used was Bootstrapping.

**TABLE 3:** Random slopes in each model.

	Testing for…	Random slopes
Model 1 and 2	H1a and H1b	Information transparency, charity preferences, amount for participants, amount for charity, and interaction term between information transparency and charity preferences
Model 3	H2a	Charity preferences, amount for participants, and amount for charity
Model 4 and 5	H2b	

### 3.1. Preliminary analyses

First, to evaluate whether gender and/or age effects were present in participants’ equal-unequal decisions while accounting for the dependency in data, two mixed-effect logistic models were fitted for prosocial and selfish decision trials. For gender, the results showed that this factor did not have a significant influence on decisions in both prosocial (odds ratio = 0.97, *p* = 0.91, 95% CI [0.59, 1.61]) and selfish trials (odds ratio = 0.93, *p* = 0.79, 95% CI [0.58, 1.52]). Similarly, age did not significantly influence decisions in both prosocial (odds ratio = 1.00, *p* = 0.82, 95% CI [0.96, 1.04]) and selfish trials (odds ratio = 0.98, *p* = 0.21, 95% CI [0.94, 1.01]). Thus, gender and age were not included as control variables in the main analyses.

Second, charity ranking was used to assess the most and the least preferrable charitable categories to be used in the experiment, and therefore a paired sample *t*-test was conducted to ensure the most vs. the least charitable categories differ in their ratings of personal importance. The result showed that participants’ ratings differ significantly (*t*(114) = 14.68, *p* < 0.001) between the most (*M* = 6.47) and the least charitable categories (*M* = 4.16).

### 3.2. Information transparency and donation behaviors

We examined whether participants behaved differently in the complete vs. incomplete information conditions (H1a), and with the most vs. least preferred charitable categories (H1b). Information transparency (Table 4) had no significant effect on participants’ choices between equal or unequal options in both selfish (Model 1; odds ratio = 1.29, *p* = 0.18) and prosocial trials (Model 2; odds ratio = 0.84, *p* = 0.41). Charity preferences also did not significantly influence equal-unequal decisions in selfish (Model 1; odds ratio = 2.49, *p* = 0.06) and prosocial trials (Model 2; odds ratio = 0.68, *p* = 0.17). Thus, H1a and H1b were not supported.^4^

**TABLE 4:** Mixed-effect model results for selfish and prosocial trials in the experiment.

	Model 1: Selfish trials (*n* = 2280)		Model 2: Prosocial trials (*n* = 2280)	
	**Odds ratio**	**95% CI**	**Odds ratio**	**95% CI**
Intercept	0.83	[0.33, 1.94]	0.87	[0.38, 2.01]
Conditions	1.29	[0.83, 1.96]	0.84	[0.54, 1.37]
Charity preferences	2.49	[0.96, 9.71]	0.68	[0.36, 1.31]
Amount for participant	1.09	[0.78, 1.53]	1.03	[0.75, 1.44]
Amount for charity	1.17	[0.82, 1.65]	1.43**	[1.11, 1.85]
Conditions × charity preferences	0.84	[0.40, 1.77]	1.11	[0.59, 2.10]
Pseudo *R*^2^	0.01		0.01	

**p < 0.01. Grouping variable = Participants (*N* = 114).

The amount allocated to charity was significant in predicting equal-unequal decisions in prosocial trials (Model 2; odds ratio = 1.43, *p* = 0.001). Thus, the odds of selecting the unequal option increased by 43% (1.43 – 1) when the amount allocated to charity increased by one coin. In other words, participants were more likely to choose the option that benefited a charity when the amount increased.

**FIGURE 2:**
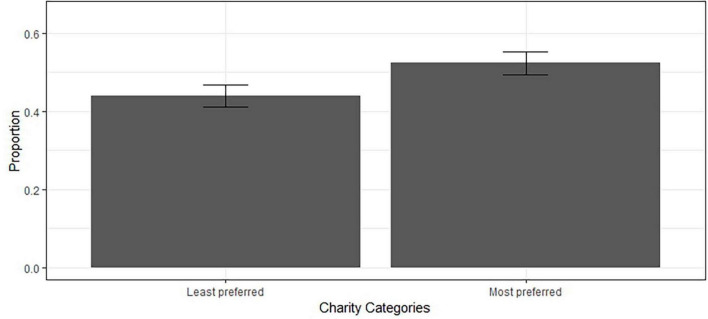
Bar chart (with error bars) illustrating the proportion of participants that choose to seek full information when deciding for their least preferred and most preferred charitable organization categories.

For the incomplete information condition, we first examined whether information seeking behavior was influenced by charity preference (H2a). In Model 3, participants’ charity preference significantly predicted their information seeking behavior (Figure 2 and Table 5; odds ratio = 2.28, *p* < 0.001). The contrast showed that the odds for participants to reveal the exact allocation increased by 128% (2.28 – 1) when the trial concerned their most preferred charitable category compared to the least preferred. Thus, H2a was supported.

**TABLE 5:** Mixed-effect model results for all trials in incomplete information condition.

	Model 3: All trials from incomplete information condition (*n* = 2280)	
	**Odds ratio**	**95% CI**
Intercept	1.06	[−0.56, 0.69]
Charity preferences	2.28***	[0.46, 1.24]
Amount for participant	0.99	[−0.20, 0.20]
Amount for charity	0.99	[−0.19, 0.16]
Pseudo *R*^2^	0.01	

***p < 0.001. Grouping variable = Participants (*N* = 114).

Then, among the incomplete information trials, we examined whether information seeking behavior (i.e., to reveal or not reveal the full distribution) influenced donation decisions (H2b). Table 6 showed that information seeking behavior was not a significant predictor of participants’ donation decision for both selfish trials (Model 4; odds ratio = 1.05, *p* = 0.77) and prosocial trials (Model 5; odds ratios = 1.01, *p* = 0.96). Thus, H2b was not supported.

**TABLE 6:** Mixed-effect model results for selfish and prosocial trials in incomplete information condition.

	Model 4: Selfish trials in incomplete information condition (*n* = 1140)		Model 5: Prosocial trials in incomplete information condition (*n* = 1140)	
	**Odds ratio**	**95% CI**	**Odds ratio**	**95% CI**
Intercept	0.89	[−1.03, 0.73]	0.65	[−1.68, 0.63]
Charity preferences	2.43	[−0.04, 2.35]	0.79	[−0.99, 0.58]
Amount for participant	1.04	[−0.28, 0.38]	1.24	[−0.16, 0.67]
Amount for charity	1.22	[−0.12, 0.50]	1.38	[−0.04, 0.70]
Information seeking	1.05	[−0.28, 0.37]	1.01	[−0.33, 0.34]
Pseudo *R*^2^	0.01		0.01	

Grouping variable = Participants (*N* = 114).

## 4. Discussion

The primary objective of this study was to investigate whether situational characteristics would lead to willful ignorance, and thus, in effect, influence prosocial (or self-benefiting) donation behaviors in a binary DG. However, despite the seemingly robust effect of willful ignorance found in university laboratories (e.g., Dana et al., 2007; Larson and Capra, 2009; Matthey and Regner, 2011; Feiler, 2014), we found no evidence of willful ignorance in this online study, nor observed a decrease in prosocial decisions. Results across the mixed-effect models suggested that information transparency (complete vs incomplete information condition; H1a) and information seeking behavior (reveal vs. not reveal donation distribution; H2b) did not significantly predict donation decisions. In fact, regarding charity preference, it indicated an opposing effect on donation decisions (H1b), in which participants were less generous with their most preferred charitable category. Nonetheless, the findings supported the relationship between participants’ charity preferences and information seeking behaviors (H2a).

### 4.1. The absence of willful ignorance

The results here are in striking contrast to those of Dana et al. (2007) and relevant experimental replications. Willful ignorance, and the subsequent decrease in selecting the prosocial option in the incomplete information condition, was a consistent finding across these previous studies, however, they were not evident in the present sample. We believe that the situational characteristics of the experiment might contribute to this outcome. Examining social interaction broadly, the situational strength hypothesis proposes that in strong situations, behavioral expressions are not directed by personality differences but by the perceived situation. But in weak situations, individual differences in behavioral expressions are more dominant (Mischel, 1977)^5^.

The present experiment was designed such that the complete information condition was intended to constitute a “strong” situation as participants might act in correspondence with social desirability, whereas the incomplete information condition would blur the situational demands to form a weak situation. However, given the online platform employed here – which might actually better model many real-life donation situations – both social pressures and behavioral norms are absent when anonymity is guaranteed. Therefore, in this absence of social and/or moral obligation, participants were likely not particularly motivated to remain ignorant about the payoff information to charity.

With reference to the distribution of donation decisions in complete and incomplete information conditions (Table 2), we speculate that the situational strength across conditions did not change, given that the choice proportions of the selfish and prosocial options were similar in the two conditions. *Post hoc* examination of the proportions of choosing the unfair options in this study, contrasting with Dana et al. (2007) at the baseline, point to differences between online and laboratory experiments in responses (see Supplementary Appendix 2). It suggests that the self-serving behaviors were already high at the baseline in the online experiment, thus the room for increase shrinks even when incomplete information afforded an excuse to act selfishly in willful ignorance.

Some studies question whether online and face-to-face setting would evoke similar responses, and there are inconsistent findings. For example, while a meta-analysis found no obvious differences in social desirability scores across the means of data collection (Dodou and de Winter, 2014); another showed that depending on the nature of the questions (e.g., attitudes/behaviors that are sensitive or with negative valence), different survey methods affect responses (Zhang et al., 2017). Donation behavior is a value-laden action that could influence self-perception and social reputation, which raises important questions regarding how experimental studies are conducted in an age of online charitable appeals and giving, and whether the perceived anonymity of online choices may be fundamentally different from those carried out in person.

### 4.2. Inconsistent findings with charity preferences in decision-making and information seeking behaviors

The effect of charitable preferences was not observed in participants’ monetary allocation decisions, but was found in their information seeking behaviors. The results suggest that people’s information seeking behavior is driven by social preference, while actual decisions themselves could be influenced by other factors. For example, direct material gain or the psychological benefits derived from one action over the other could have a greater influence on actual donation behavior (Bekkers and Wiepking, 2011). A point to note when interpreting the results is that the personal importance ratings for all charities were positively skewed. This implied that, in general, participants thought that the five social causes presented were meaningful and held in positive regard (even for the least preferred charity). Though the most and the least preferred charitable categories were significantly distinct, the least preferred charity could also fall within the moral circle of participants. Then, they might have been intrinsically motivated to act prosocially toward all charitable categories in the experiment.

Alternatively, when considering the other perspective in the ingroup favoritism literature – the Bounded Generalized Reciprocity (BGR; Yamagishi and Kiyonari, 2000; Balliet et al., 2014), which complements Social Identity Theory (Tajfel, 1978, 1982), they tap into different psychological processes that explain intergroup behaviors. In particular, BGR takes into account the role of interdependence of interest (Yamagishi and Kiyonari, 2000), specifying that ingroup favoritism would be intensified when the outcome of the actors are interdependent. In our experiment, the outcome of participants was not dependent on the charity which they donated to. Thus, donation behaviors did not differ between the most and the least preferred charitable categories perhaps due to the absence of ingroup favoritism.

It is also possible that participants underwent a dual-route decision-making process when confronted with charities with different levels of personal importance. Specifically, given that there are many types of prosocial behaviors that people can engage in with charities (apart from making monetary donations), it is possible that a person may regard (small) donations as a relatively low-commitment activity. Instead, as with volunteering or organizing promotional campaigns, a person may regard activities that involve investments of personal time, energy, and competencies (e.g., knowledge and skills) as reflective of greater personal commitment. The premise here is that the behavioral commitments to the groups we care about is proportional to the degree of preference we have for them. Indeed, when it comes to donating money vs. donating time (i.e., volunteering), different determinants are involved (Lee and Chang, 2007, 2008). Monetary donation is mostly driven by extrinsic motivations, such as income or age; while volunteering is intrinsically driven by psychographic or attitudinal-based factor (Lee and Chang, 2008). Social preference for different charitable causes lead to intrinsic motivations as people genuinely favor certain population as the recipients of their resources. Regardless of the efficiency of utilizing resources, people generally have a stronger desire to donate time and effort than to donate money for the charity they selected (Brown et al., 2019). Thus, the monetary donation option in this study might offer an alternative to support good causes without spending much personal resources for their least preferred (but still meaningful) charitable category. All in all, this counter-intuitive result might hint at a dual-process approach to understanding prosocial behaviors toward charities of different personal preferences.

### 4.3. Strengths and significance

The study offers a theoretical implication. The self-signaling model (Bodner and Prelec, 2003) neglects contextual influence on behaviors. As suggested by Dana et al. (2007) and replicated studies (e.g., Larson and Capra, 2009; Matthey and Regner, 2011; Feiler, 2014), willful ignorance could be a strategy adopted by individuals to avoid moral dilemmas when having to choose between material gain or social desirability. However, the results of this study call for a re-examination of these seemingly robust conclusions. Specifically, this could act as a starting point to better understand what situational characteristics are essential and necessary for willful ignorance to occur.

The current study serves as a useful addition to the literature on prosocial behavior, such as those conducted via monetary donation. First, contrary to majority of previous replications of Dana et al. (2007), this study was conducted online, demonstrating that the effect of willful ignorance may be highly context dependent. Second, the study recruited a more representative, non-student sample, with various age groupings. This allows for a more comprehensive examination of the previous observed effect with the use of the binary DG paradigm.

### 4.4. Limitations and future directions

First, while our experiment was not designed to further explore reasons for the absence of willful ignorance, the results make us suspect that situational strength can be a contributing factor. Further research could manipulate the strength of experimental situations in both lab and online environments to scrutinize the necessary context for the effect of willful ignorance to occur. Moreover, other than the medium used in conducting the experiment, one’s perception of the situation could also affect situational strength. In order to understand the rationale behind social decisions, future studies could probe participants with their fairness perceptions or inquire *post hoc* reasoning for their general decision rule used when choosing equal or unequal distribution of the resources.

Second, the scope of available charitable categories for participants to rank was rather limited. In order to further examine whether there is a dual-process in prosocial decision-making, it would be helpful to include a wider range of charitable categories in future studies. Then, the studies can experiment with charities that vary in distance to the self in the moral circle, and perhaps even include charitable causes that are actively aversive to participants. In addition, they could also examine various prosocial behaviors other than pure monetary donation, for example, future studies could assess willingness to volunteer or post supportive messages on social media.

## 5. Conclusion

In this study, we extend understanding of donation behaviors in the context of an online binary DG. In contrast to previous laboratory studies, we found no differences in donation behaviors between situations of complete and incomplete information. Thus, there was no support for the effect of willful ignorance in the online context. Importantly, our results indicated that participants’ information seeking behavior was driven by their social preferences for charities; however, that did not translate into more generous donation decisions for their most preferred charitable category, signaling a divergent motivation between information seeking and actual decision behavior.

## Data availability statement

The datasets presented in this study can be found in online repositories. The names of the repository/repositories and accession number(s) can be found below: https://osf.io/73h5q/.

## Ethics statement

The studies involving humans were approved by the Ethics Committee Faculty of Social Sciences, Behavioral Science Institute, Radboud University Nijmegen. The studies were conducted in accordance with the local legislation and institutional requirements. The participants provided their written informed consent to participate in this study.

## Author contributions

MS: Conceptualization, Formal analysis, Investigation, Methodology, Project administration, Writing – original draft. AS: Resources, Supervision, Writing – review and editing.

## Footnotes

1 A United Kingdom-based online participant recruitment platform.

2 Pound Sterling is the default currency of the platform.

3 A coin-flip task was included in data collection to explore aggregate cheating behaviors under anonymity, which address another set of hypotheses from the current manuscript. The task recorded the result of the two coin-flips (i.e., heads or tails) reported by participants.

4 *Post hoc* analyses were carried out and found that donation decisions of participants (i.e., the proportion of choosing equal vs. unequal distribution of resources) also did not differ between the two levels of charity preferences, across inequality levels (see Supplementary Appendix 3).

5 According to Mischel (1977), the strength of situations is determined by four conditions: (1) common construal of situations, (2) uniform expectancy of demanded behaviors; (3) incentives or motivation to perform such behaviors; and (4) skills or abilities to perform such behaviors.
