# Supplementary material for: An experimental study of information transparency and social preferences on donation behaviors: the self-signaling model
Source: Front Psychol. 2023 Nov 10;14:1258808. doi: 10.3389/fpsyg.2023.1258808 (PMC10667727; doi:10.3389/fpsyg.2023.1258808)
Supplement: Supplementary file 1 [file Table_1.pdf]

Appendix 1
The Walk-through Example of the Incomplete Information Block

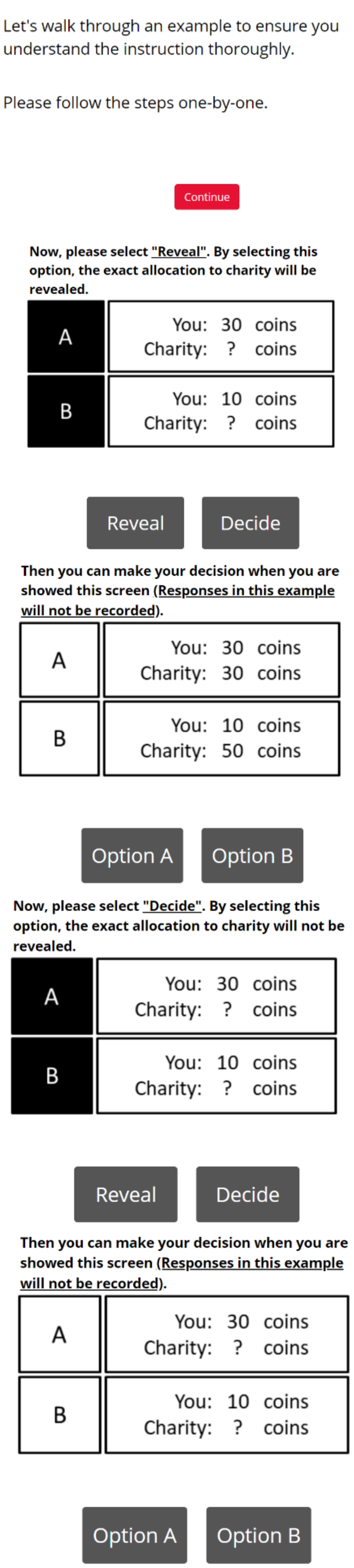

(1) Introduction screen of the walk-through

(2) Illustrating the 'Reveal' option, with explantion

(3) Demonstrating the consequence of selecting 'Reveal' – showing the exact distribution for charity

(4) Illustrating the 'Decide option, with explantion

(5) Demonstrating the consequence of selecting 'Decide – the exact distribution for charity remains unknown

Figure S1. Walk-through of the consequences of the 'Reveal' and 'Decide' options in the incomplete information blocks

Appendix 2
Compare proportions of choosing the unfair option in baseline with Dane et al. (2007)

Based on the recommendation of the reviewer, we conducted additional analyses to examine whether participants felt increased anonymity in online experiment. We compared our proportion of choosing the unfair option in baseline condition with that in Dane et al. (2007). As we did not use the exact same self-to-other distribution ratio, we compared each of the trials that consisted of a larger distribution to the self and a smaller distribution to others, with the single trial in Dane et al. (2007). The statistical tests were carried out with the online calculator[1].

The results showed that, participants in our online study chose significantly more the unfair options at all trials referencing to their most preferred charitable category and two trials referencing to their least preferred charitable category, compared to Dana et al. (2007). It points to the direction that participants in this online experiment has a higher proportion in choosing the unfair option, in contrast to laboratory experiment. The increased felt anonymity offered by the online environment could be one factor contributing to such difference.

Table S1. Proportion of choosing the unfair option in baseline, comparing with Dana et al. (2007)

Self-to-Charity Distribution	Most preferred charitable category			Least preferred charitable category		
	% choosing unfair option	Chi-Square		% choosing unfair option	Chi-Square	
		X^2	*p*		X^2	*p*
10:0	54/114 (47%)	5.07	.02	55/114 (48%)	5.44	.02
9:1	58/114 (51%)	6.65	.01	49/114 (43%)	3.45	.06
8:2	59/114 (52%)	7.09	.01	50/114 (44%)	3.74	.05
7:3	59/114 (52%)	7.09	.01	46/114 (40%)	2.64	.10
6:4	57/114 (50%)	6.23	.01	51/114 (45%)	4.05	.04
Overall	287/570 (50%)	4.23	.04	251/570 (44%)	2.42	.12

Note. In Dana et al. (2007), 5 out of 19 participants (26%) chose the unfair option (i.e., 6 for self and 1 for other) in the baseline condition.

[1] https://www.medcalc.org/calc/comparison_of_proportions.php

Appendix 3

Comparing proportions of choosing the unfair option across the two charity preferences, at different inequality levels (differences in self-to-charity distribution)

Table S2. Proportion of choosing the unfair option in baseline

Differences in self-to-charity distribution (self – charity)	Most preferred charitable category	Least preferred charitable category	Chi-Square#	
	% choosing unfair option	% choosing unfair option	X^2	*p*
Baseline				
10 (10 – 0)	54/114 (47%)	55/114 (48%)	0.00	1.00
8 (9 – 1)	58/114 (51%)	49/114 (43%)	1.13	.29
6 (8 – 2)	59/114 (52%)	50/114 (44%)	1.13	.29
4 (7 – 3)	59/114 (52%)	46/114 (40%)	2.54	.11
2 (6 – 4)	57/114 (50%)	51/114 (45%)	0.44	.51
-2 (4 – 6)	54/114 (47%)	56/114 (49%)	0.02	.89
-4 (3 – 7)	52/114 (46%)	61/114 (54%)	1.13	.29
-6 (2 – 8)	60/114 (53%)	61/114 (54%)	0.00	1.00
-8 (1 – 9)	61/114 (54%)	62/114 (54%)	0.00	1.00
-10 (0 – 10)	63/114 (55%)	63/114 (55%)	0.00	1.00
Incomplete Information				
10 (10 – 0)	56/114 (49%)	54/114 (47%)	0.02	.89
7 (9 – 2)	59/114 (52%)	57/114 (50%)	0.02	.89
6 (8 – 2)	56/114 (49%)	51/114 (45%)	0.28	.60
6 (7 – 1)	59/114 (52%)	52/114 (46%)	0.63	.43
2 (6 – 4)	64/114 (56%)	55/114 (48%)	1.13	.29
-4 (4 – 8)	53/114 (46%)	60/114 (53%)	0.63	.43
-4 (3 – 7)	55/114 (48%)	61/114 (54%)	0.44	.51
-1 (2 – 3)	53/114 (46%)	52/114 (46%)	0.00	1.00
-8 (1 – 9)	60/114 (53%)	58/114 (51%)	0.02	.89
-4 (0 – 4)	46/114 (40%)	52/114 (46%)	0.45	.50

Note. # df = 1
